# Measurement Method of Fuel Nozzle Cone Angle Based on Point Cloud Slicing

**DOI:** 10.3390/mi17060706

**Published:** 2026-06-09

**Authors:** Yeni Li, Zusheng Lin, Xiaodong Tang

**Affiliations:** College of Mechanical and Automotive Engineering, Xiamen University of Technology, Xiamen 361024, China

**Keywords:** cone angle measurement, point cloud preprocessing, point cloud slicing, circle fitting, RC-RANSAC

## Abstract

To address the issues of low efficiency and large errors in traditional dimensional measurement strategies for fuel nozzles, this paper proposes an improved region-constrained Random Sample Consensus (RANSAC) circle fitting method for high-precision measurement of the inner hole cone angle. Three-dimensional point clouds are extracted using a shape-from-focus method. The point cloud slices are then projected onto a two-dimensional plane, and the slice edges are extracted. Based on the edge shape distribution, the candidate point selection strategy of RANSAC is optimized: the initial circle is divided into eight sector regions, and three points are randomly selected from three distinct regions to fit candidate circles. After multiple iterations, the optimal fitting circle is obtained. A comparative analysis is conducted among the least squares method, standard RANSAC, and the proposed algorithm, with three quantitative metrics—residual standard deviation (σ), root mean square error (RMSE), and inlier ratio (ε)—introduced to evaluate the fitting quality. Experimental results show that the proposed region-constrained RC-RANSAC method achieves the best performance among the three, yielding σ = 2.826 px, RMSE = 2.826 px, and ε = 95.2%, and attains a cone angle deviation of only 1.0°, which closely agrees with Keyence ultra-depth measurements (error 0.8°). This method provides a new approach for accurate and robust cone angle measurement of fuel nozzle inner holes.

## 1. Introduction

The fuel nozzle’s spray performance has a great impact on the efficiency of the engine. In the combustion chamber of an aircraft engine, the droplet is mixed with air to form an air-mixed fuel, which can provide energy for full combustion. Common engine fuel nozzles in the combustion chamber include the single-orifice swirl atomizer and the dual-orifice swirl atomizer. The dual-orifice swirl atomizer’s structure is relatively complex, mainly composed of an outer swirl injector and an inner swirl injector, which can provide fuel for the main oil circuit and the vice oil circuit. The dual-channel centrifugal nozzle is recognized for its optimal atomization performance and is widely used to meet varying fuel supply demands. This nozzle mainly consists of a nozzle with a gas hood, an auxiliary nozzle, and a swirler. The machining quality of the fuel nozzle directly affects the spray cone angle, flow rate, and uniformity of distribution within the combustion chamber.

The auxiliary nozzle of the fuel injector features typical characteristics such as an inner conical hole, a threaded hole, and a swirling groove. Additionally, the surface roughness of the inner bore needs to attain a level of 0.1–0.4 μm. Common methods for measuring micro-holes can be categorized into two main types: contact and non-contact measurements. Contact measurement techniques primarily include internal micrometers, calipers, coordinate measuring machines (CMMs), and profile measuring instruments [1]. Among these, the CMM method allows for precise measurement of circular holes [2], obtaining key parameters such as the center and radius; however, it requires a high degree of equipment stability and is not well-suited for online measurements. In contrast, non-contact measurement methods encompass microscopy-based visual measurements [3], white light interferometric microscopy, atomic force measurement, CT measurement [4], and laser measurement techniques [5]. Given that traditional contact measurement methods struggle to meet the demands for micrometer-level detection and have a certain degree of destructiveness, their applicability in high-precision inspection fields has diminished. Consequently, non-contact measurement methods, particularly optical measurement techniques, are increasingly being employed for measuring small holes. Renaud et al. [6] uses the method of tomography to detect the discharge holes in the combustion chamber. It can measure 60 holes within 15 min, and the deviation of drilling angle detection is between 0.25° and 0.55°. It provides quality control guidelines to optimize the laser drilling process. Chen et al. [7] adopts the SFF method for deep hole measurement and proposes a volume neighborhood weighted interpolation strategy, which can accurately locate sub-voxels on the measured surface and improve the accuracy of inner hole measurement. Micro-hole measurement methods based on machine vision can extract comprehensive dimensional information and morphological features, demonstrating high efficiency and accuracy. In the literature regarding the measurement of circular holes, researchers have proposed various methods to achieve high-precision measurements. These techniques can be broadly categorized into two types: (1) enhancing the edges of the micro-hole to improve the localization and recognition of circular holes [8]; (2) developing algorithms for circle fitting to enhance the measurement accuracy [9]. In the first type of method, the main approach is to enhance the accuracy of edges through image preprocessing. Tao et al. [10] proposes a robust high-precision circular hole reconstruction method based on multi-camera systems, which obtains ellipses through epipolar geometric matching and optimizes them. Qiu et al. [11] proposed a novel focusing measurement operator based on grayscale difference and adaptive neighborhood size, which can better reconstruct and measure three-dimensional point clouds. Truong et al. [12] proposed an automatic machine vision system and deep learning segmentation model in detecting circular hole edge boundaries and cracks. Sheng et al. [13] proposed a reconstruction method based on reflection characteristics for measuring defect contours and the depth of the inner surface of the hole.

The second type of method mainly involves the circle fitting algorithm to improve the measurement accuracy of circles. Circle fitting methods include traditional methods and improved methods. The traditional methods mainly include the least squares method, Hough transform method, random consistency sampling method, etc. Xu et al. [14] proposes a robust circle detection algorithm based on the least squares fitting method assisted by the radius of the arc chord region. Huang et al. [15] researched a technique based on neural networks and image processing for detecting micro-nozzle inner hole defects, using the Circle Inspection algorithm to achieve micro-nozzle inner hole circle measurement. Michalowska et al. [16] proposed a method that combines the Hough transform denoising process and robust least squares circle fitting to effectively extract tree slice circles from laser-scanned point clouds. Antoniadis et al. [17] used RANSAC and Iterative Reweighted Total Least Squares (IRTLS), combined with Nelder Mead’s method, to optimize the fitting of circles and cylinders. Jiang et al. [18] proposed an improved random Hough transform circle detection algorithm that optimizes the accuracy of candidate circle fitting.

Microscopic vision measurement based on focused morphology recovery utilizes the small depth of field of the lens to detect depth. The maximum focus amount under each window is calculated using the focus measure operator, which corresponds to the initial depth of the sequence image frame rate. Based on this principle, the initial depth values of all windows in the entire image are extracted to obtain the three-dimensional point cloud. The three-dimensional point cloud measurement method is more comprehensive and accurate than two-dimensional image detection. In order to improve the accuracy of point cloud extraction, researchers have been committed to improving the focused morphology restoration technique in the following aspects: optimization of depth maps [19,20], adaptive window size [21], focus measure operator [22], interpolation algorithm to obtain continuous depth values [23], using traditional calculation methods [24], as well as deep learning classification extraction methods [25]. Both methods are aimed at improving the accuracy of point cloud extraction for subsequent measurements of size or roughness.

This paper proposes a dimensional detection method based on shape from focus (SFF) to meet the requirements of the cone hole in fuel nozzles. The method extracts high-precision point clouds through an optimized focus measure operator, and then combines radius filtering and statistical filtering to remove redundant points from the point cloud data. Finally, by comparing and analyzing the three circle fitting methods, the results show that the improved robust RANSAC circle fitting method has the smallest deviation with 1° in circle fitting.

The structure of the paper is as follows: Section 2 provides a detailed description of point cloud preprocessing and circle fitting methods, while Section 3 compares and analyzes the performance of circular fitting algorithms; Section 4 compares three circle fitting algorithms and discusses the advantages, limitations, and future improvements of the proposed RC-RANSAC. Section 5 draws certain conclusions. This non-contact measurement method based on micro-vision can accurately measure conical and circular holes, and has certain application value.

## 2. Materials and Methods

**Figure 1:**
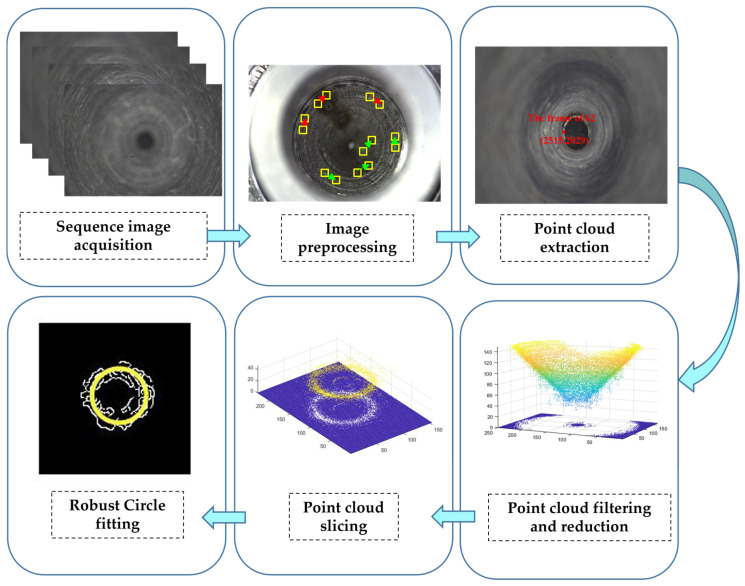
Point cloud extraction and circle fitting diagram (The arrows indicate the six steps from sequence image acquisition to circle fitting).

As illustrated in Figure 1, the architecture of the proposed 3D measurement and circle detection algorithm is divided into six stages, which are sequence image acquisition, image preprocessing, extracting point cloud, point cloud filtering and reduction, point cloud slicing and robust circle fitting.

### 2.1. Image Acquisition and Point Cloud Extraction

**Figure 2:**
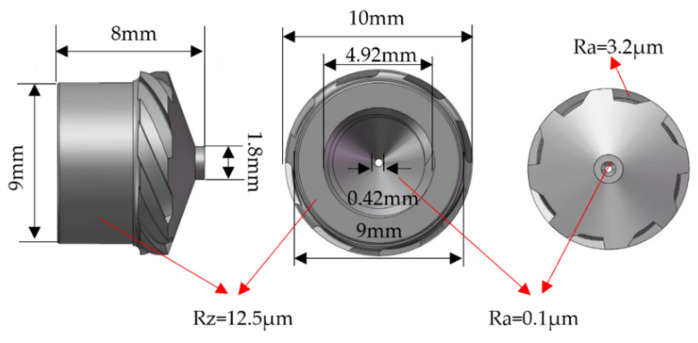
Requirements for dimensions and morphology of key positions of engine fuel nozzle.

Twenty-four fuel injectors are uniformly welded to the annular fuel pipe, atomizing the fuel and injecting it into the combustion chamber to provide power for engine operation. The fuel nozzle can achieve sufficient and uniform atomization of the fuel. The key dimensions and morphological characteristics of fuel nozzles directly determine the economic performance of the combustion chamber and the conversion efficiency of fuel. As shown in Figure 2, the dimensions and morphology indicators of key positions of the fuel nozzle include the depth and width from the bottom of the swirl groove to the center hole, the quality of the inner hole surface, and the size of the inner hole cone angle. The specific steps for obtaining 3D point cloud data based on SFF technology are as follows:

First, place the precision-machined nozzle onto the inspection platform using a fixture, with the inner hole facing upward, and focus the microscope lens on the bottom of the nozzle.

Second, the initial position for image acquisition is the lowest point of the inner hole of the nozzle. The *Z*-axis moves upward in a step size of 10 µm/s. A total of 200 sequential images are acquired within a distance of 2 mm, and the *Z*-axis travel is recorded by the encoder.

Third, segment the images into a window size of 24 × 24, and compute the sharpness value for each window using an improved variance focus measure (FM) operator. By traversing the focus values of all sequential images under a given window, the maximum focus value and its corresponding frame number are obtained. This frame number represents the initial point cloud data.

Fourth, the microscope calibration scale is used to determine the field of view size along the XY axis, ignoring minor deviations. Then, the pixel equivalent of the *Z*-axis is calculated based on the travel distance. Finally, the initial point cloud data is converted into real 3D point cloud data.

### 2.2. Outiler Removal and Down Sampling

The three-dimensional point cloud extracted based on the SFF principle contains some redundant or noise points. Platform vibrations, changes in the camera’s field of view during the *Z*-axis movement, FM operator, and window size can all contribute to noise points [26]. Filtering and point cloud simplification can improve the measurement accuracy of the point cloud. Methods for point cloud filtering include radius filtering, statistical outlier removal, passthrough filtering, bilateral filtering, among others [27]. Considering the characteristics of the fuel nozzle point cloud, statistical outlier filtering is applied to remove the noisy data.

**Figure 3:**
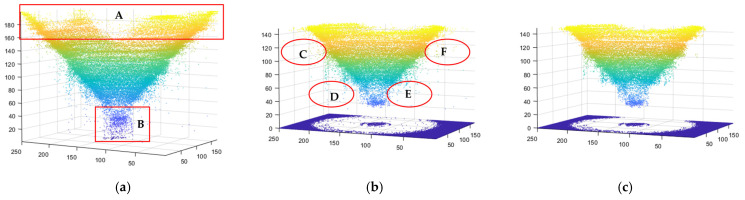
Schematic diagram of point cloud filtering. (**a**) Initial point cloud. (**b**) Capture point clouds within a certain height range. (**c**) Point cloud filtered by outlier filtering. (A is the point with a Z-axis heights exceeding 150 frames. B is the point with heights below 30 frames. C, D, E, F are the invalid points).

Due to the limited measurement information in the upper and lower parts of the acquired nozzle inlet point cloud, as shown in Figure 3a for the points corresponding to regions A and B (i.e., the points with *Z*-axis heights exceeding 150 and those with heights below 30), these points are assigned a *Z*-axis height of zero. Specifically, the point cloud in area A of Figure 3a is the inlet edge point cloud. During the collection process, due to a limited field of view, the full view of the edge mouth was not displayed; therefore, the point cloud collected in this area represents only the edge part, making the calculation of the cone angle of the inner hole invalid. The B area in Figure 3a is the point cloud of the inlet and outlet, which is a circular hole outlet with a certain height but unrelated to the cone angle of the inner hole, and thus also constitutes an invalid point cloud. Figure 3b shows the truncated point cloud, which still contains outlier data. The points in regions C, D, E, and F are invalid and can be removed using statistical outlier filtering, resulting in the filtered point cloud shown in Figure 3c.

**Figure 4:**
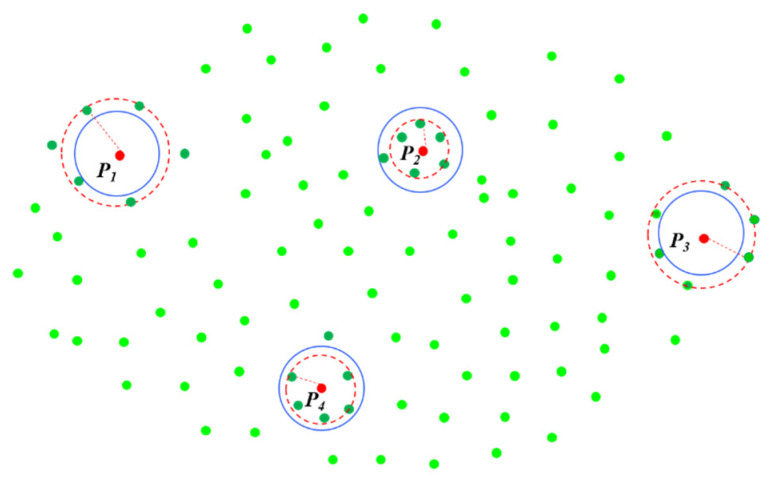
Principle of statistical outlier removal filtering (the red dashed line represents the average distance of neighboring points, while the blue solid line denotes the standard range area).

As shown in Figure 4, points P1 and P3 are outliers, while points P2 and P4 are valid points. Assuming the neighborhood size (k = 6), the average distance and standard range within the neighborhood of points P1, P2, P3, and P4 are calculated. The red dashed box represents the average distance of the (k)-nearest neighbors, while the blue solid box denotes the standard range area. If the average distance between the neighborhood points and the central point exceeds the blue solid box, the point is considered for deletion.

After filtering the point cloud, if the amount of point cloud data is large, further simplification is required. Voxel down sampling divides the point cloud into n cubic voxels and replaces all points within a voxel with the centroid or a specific point within the voxel, thereby achieving point cloud simplification. This algorithm performs better than random down sampling methods in preserving the characteristics of the point cloud. However, the centroid of the voxel may not coincide with the original point, and thus fine features cannot be guaranteed. By replacing the entire voxel with the centroid, this method is referred to as uniform voxel down sampling.

**Figure 5:**
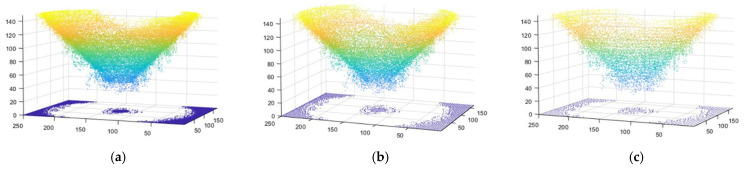
Uniform voxel down sampling for point cloud simplification. (**a**) Initial point cloud. (**b**) Grid size is 3. (**c**) Grid size is 5. (Colors are automatically mapped from low to high Z-coordinate values: yellow indicates regions with higher height, and blue indicates regions with lower height).

Based on the desired simplification effect of the point cloud, the voxel grid size is set accordingly. A larger grid size results in fewer retained points, while a smaller grid size retains more points. As shown in Figure 5a, the initial point cloud contains 40,304 points. As shown in Figure 5b, when the grid size is set to 3, the number of points decreases to 20,235. As shown in Figure 5c, when the grid size is set to 5, the number of points decreases to 8876.

### 2.3. Robust Circle Fitting Method for Cone Angle

#### 2.3.1. Calculation of Inner Hole Cone Angle

**Figure 6:**
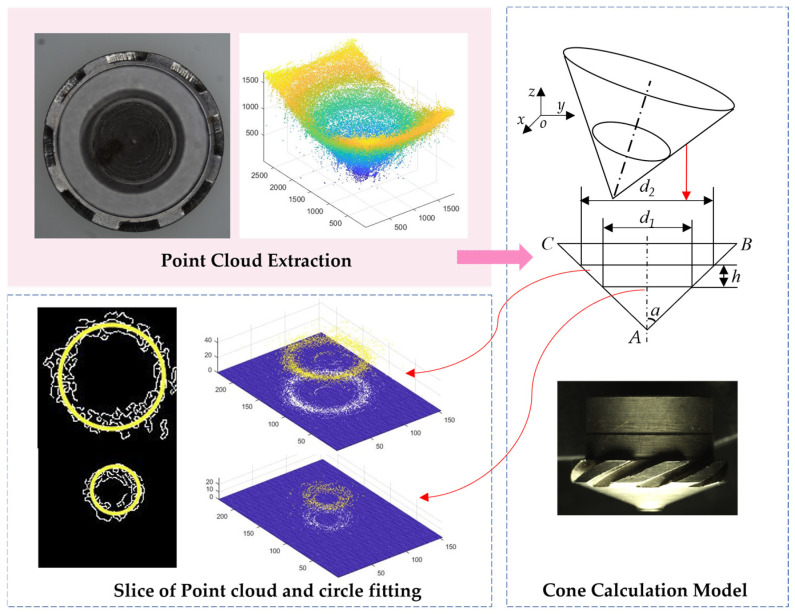
Principle of cone angle calculation. (The red arrows indicate the process of circle fitting and taper calculation from point cloud slices).

The measurement method based on point cloud slicing takes point cloud slices of a certain thickness at two different positions on the *Z*-axis [28], converts the slices into two-dimensional images, extracts edges, obtains the position of the center and the size of the radius by circle fitting [29]. According to the taper calculation principle shown in Figure 6, the taper of the nozzle inner hole is calculated.

In comparative analysis of the calculation principle of the taper, the definition of a taper is as follows:

(1) $$G=\frac{D_{2}-D_{1}}{H}$$

(2) $$\tan a=\frac{R_{2}-R_{1}}{H}=\frac{D_{2}-D_{1}}{2H}$$

The cone angle of the inner hole is:

(3) $$a=\arctan\frac{D_{2}-D_{1}}{2H}$$

In the above equation, $D_{2}$ is the large circle diameter, *D*_1_ is the small circle diameter, *a* is the cone angle, and *H* is the distance between the two cross-sections of the cone. After converting the 3D point cloud slice into a 2D image, edge extraction and circle fitting are performed. Then, according to Equations (1)–(3), the taper and cone angle of the nozzle inner hole can be calculated.

#### 2.3.2. Classic Circle Fitting Method

Classic circle fitting methods include Hough transform, least squares fitting, Random Sample Consensus method (RANSAC), and related improved algorithms. Circle Hough transform (CHT) is a classic circle detection algorithm that maps points on an image to Hough space to form a conical surface. If three points in the image are on the same circle, the three conical surfaces they form in Hough space will intersect at one point. The maximum cumulative value of the intersection points of the conical surfaces is calculated to obtain the corresponding circle parameters in the image space. This method requires a large amount of computation, occupies a large amount of storage space, and is sensitive to noise in the background, which can easily lead to deviations in circle fitting. The improved Hough transform method effectively improves the accuracy of circle fitting. Oualid et al. [30] proposes an incremental circle Hough transform method for automatic biological recognition to address the high computational complexity of CHT. This method reduces computation time and accurately locates the boundaries of the pupil and iris. Yang et al. [31] proposes an optimized Canny operator edge recognition and random Hough transform circle detection algorithm to solve the problems of low accuracy and slow speed in identifying the center coordinates of particle spheres, in order to obtain accurate center coordinates of particle systems.

The least squares fitting method is used to find the optimal fitting function for discrete points by minimizing the sum of squared errors, ensuring that the sum of squared distances between the fitted circular curve and the scattered points is minimized. The least squares fitting method has a better fitting effect on data with less noise and the best fitting effect on error data that conforms to normal distribution. If there are many outliers in the data, the deviation of data fitting will be significant. The improved least squares fitting method can avoid such problems. Xu et al. [32] proposed the regularized variants of the bias-corrected weighted LS method to fit circles from data with a weak geometrical constraint.

The Random Sample Consensus (RANSAC) algorithm is an iterative algorithm applied to data fitting and model parameter estimation [33]. Its characteristic is to estimate the best fitting curve from scattered data through hypothesis verification through random sampling. This method can also be regarded as an improved least squares fitting method. Firstly, three points are randomly selected from a set of two-dimensional pixels to determine an initial candidate circle model. The distance between each data point and the circle is calculated, and points with distances less than the set threshold are considered as inliers. The number of inliers is counted, and through multiple iterations, the best fitted circle with the most inliers is obtained. The method has strong robustness against noise and outliers, and can handle partially occluded or missing circular targets. Randomly select three points for circle fitting during multiple iterations, and determine the best fitting circle based on the number of inliers [14]. The performance of the RANSAC algorithm is affected by parameters such as the maximum number of iterations and distance threshold, so the values of each circle fitting are also different.

#### 2.3.3. Improved RANSAC Circle Fitting Method

When there are machining defects on the inner wall of the conical hole or factors such as vibration during the collection process, there may be some interference points or outliers in the extracted 3D point cloud data. After point cloud slicing and binarization, there may be some deviation points at the edges. If random points are directly used for circle fitting, the accuracy will be affected.

**Figure 7:**
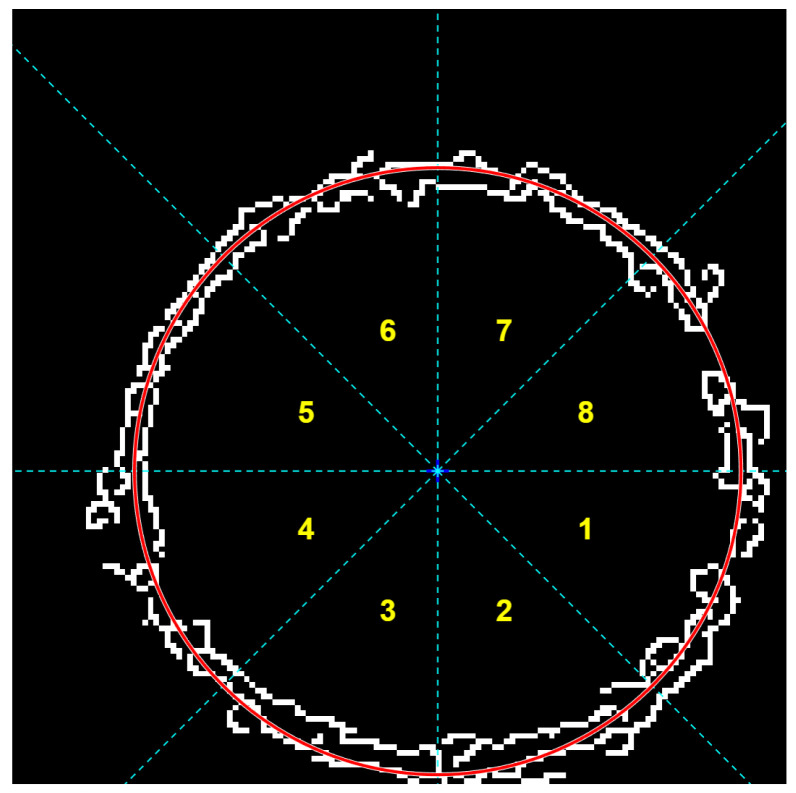
Schematic diagram of region division. (The red line represents circle fitting, the number represents eight partitions, the blue line represents the partition).

In the traditional RANSAC algorithm, random sampling often leads to uneven distribution of sample points. To address this issue, this paper proposes a sector-based division of edge points obtained after binarization of point cloud slices. The number of sectors can be set arbitrarily. Figure 7 shows a schematic diagram of sector division, where the number of sectors is set to eight. This ensures that each sector contains a sufficient number of edge points under the given distribution. Through comparative analysis of circle fitting in different divided regions, it was found that if there are too few sectors, errors caused by local deviations may arise; if too many sectors are used, the computational cost increases without significant improvement in fitting accuracy. This method, termed region-constrained RANSAC (RC-RANSAC), ensures that sample points are distributed around the entire circumference, thereby improving the accuracy of circle fitting.

The improved point selection strategy adopts the following steps:

Step 1: Randomly select three edge points for initial circle fitting, obtain the fitted center and radius parameters, and the initial circle can be used for subsequent sector area division.

(4) $$x^{2}+y^{2}+Ax+By+C=0$$

(5) $${\left(x+\frac{A}{2}\right)}^{2}+{\left(x+\frac{B}{2}\right)}^{2}={\left(\frac{A}{2}\right)}^{2}+{\left(\frac{B}{2}\right)}^{2}-C$$

(6) $$(x_{0},y_{0})=(-\frac{B}{2},-\frac{C}{2}),\;r_{0}=\frac{\sqrt{A^{2}+B^{2}-4C}}{2}$$

Step 2: Using the center of the initial circle as the pole, divide the entire circumference into 8 fan-shaped regions uniformly according to the polar angle.

Step 3: Use uniform sampling to construct candidate models, randomly select 3 different regions from 8 sector regions each time, and randomly select one point from each of these three regions as the representative point for that region.

Step 4: Fit the selected 3 points to form a candidate circle.

Step 5: Calculate the distance from all data points to the candidate circle as shown in Equation (7). Count the number of interior points with a distance less than the set point threshold.

(7) $$d_{i}=\sqrt{{\left(x_{i}-x_{0}\right)}^{2}+(y_{i}-y_{0}{)}^{2}}$$

Set a distance threshold, with points below the threshold set as inliers and points above the threshold set as outliers.

Step 6: Record the candidate model with the highest number of inliers.

Step 7: Repeat steps 3–5 until the maximum number of iterations is reached or a sufficiently good model is found, then use the least squares method for circle fitting to obtain the final circle.

Due to the deviation in image acquisition and point cloud extraction, the point cloud slice has a certain thickness, which may result in many edge lines during point cloud slicing and dimensionality reduction image processing. Some edge lines are very dense, while others are missing. If the traditional least squares fitting method is used, there will be significant deviation in circle fitting. This algorithm avoids fitting bias caused by locally clustered or sparse points, forces point selection from different regions, ensures that sampling points can cover the entire circumference of the circle, improves fitting accuracy, and avoids the influence of outliers, enhancing robustness. This method improves the point selection strategy by guiding sampling through geometric constraints, significantly enhancing the stability and accuracy of RANSAC in circle fitting.

## 3. Experimental and Analysis

**Figure 8:**
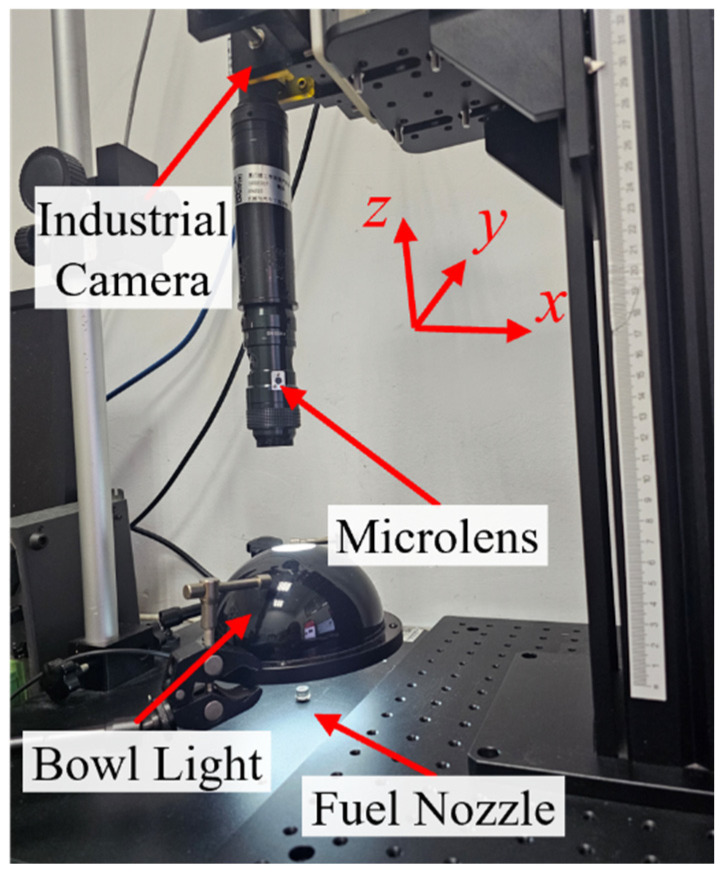
Hardware system.

Experimental environment: The processor is Intel Core i7-12700-2.10 GHz, the memory is 64 GB, and the operating system is Windows11 Professional Edition. The industrial camera is a Daheng MER-2000-19U3C (Daheng Group, Inc., Beijing, China) with a resolution of 5496 × 3672, and the microscope lens model is OPTEM-304310 (Qioptiq, Inc., NewYork, NY, USA). Its object distance is 89 mm, depth of field is between 120 and 840 mm, and magnification is 0.7×–4.5×. The upper computer uses the Matlab2020 software platform, and the lower computer uses the Delta DVP-12SA211T (Delta Electronics, Inc., Taiwan, China). The test was conducted in a laboratory environment, and the actual parts are shown in Figure 8. During the experiment, the upper computer sent instructions to the PLC, which then controlled the *Z*-axis of the screw to move from bottom to top. A bowl-shaped light source to ensure stable illumination and no shadow interference. The calculation of the cone inner hole angle is divided into two steps:

First, the RANSAC method, the least squares method, and robust RANSAC method are used to calculate the cone angle of the inner holes of fuel nozzle No. 1 and No. 2’s workpieces.

Second, a certain thickness of the fuel nozzle inlet slice point cloud is converted into a two-dimensional edge image. The improved Canny operator is used to locate the edge of the circular hole to obtain the edge points of the image. Then, the edge coordinates are fitted with the circle fitting method to obtain the center coordinates and radius. The accuracy of the circle fitting method is the key step to measuring taper. In order to facilitate the analysis of circle fitting, it is necessary to convert the pixel values of the detected circle radius into actual values. The specific formulas for calculating the relevant values are as follows:

(8) Xc=kx×w×Xi

(9) Yc=ky×w×Yi

(10) Zc=kz×w×Zi

(11) D=K×d=kx×w×d=2kx×w×R

Among them, *Xc* is the actual coordinate after *X*-axis calibration, *Xi* is the initial pixel count on the *X*-axis, *kx*, ky, kz is the pixel equivalent, *w* is the window size, *K* is the calibration coefficient, *Yc* is the actual coordinate after *Y*-axis calibration, *Yi* is the initial pixel count on the *Y*-axis and *Zi* is the initial image frame rate. Calculate the pixel equivalent *kz*, which is 10 μm for the *Z*-axis. *D* is the calibrated fitting circle diameter, d is the uncalibrated diameter, H is the real distance of two cross sections and r is the uncalibrated circle radius.

When the magnification of the microscope lens is 3× and the field of view is 4.31 mm × 2.84 mm, the pixel equivalent is:

kx=ky=4.31mm/5496 pixels=0.784 μm

Therefore, the window size w is 24, according to Equation (11); the calibration coefficient K is calculated as follows:

K=kx×w=0.784×24 μm=18.816 μm

The diameter of a circle is D:

D=K×d=kx×w×d=18.816×d

According to Equation (1), the taper is:

$$Taper=\frac{D_{2}-D_{1}}{H}$$

According to Equation (2):

$$\tan a=\frac{D_{2}-D_{1}}{2H}=\frac{R_{2}-R_{1}}{H}$$

According to Equation (3), the taper angle is β:

$$a=\arctan(\frac{D_{2}-D_{1}}{2H})=\arctan(\frac{R_{2}-R_{1}}{H})$$

(12) $$\beta =2a=2\arctan(\frac{R_{2}-R_{1}}{H})=2\arctan kx\times w\times (\frac{r_{2}-r_{1}}{100})=2\arctan18.816\times (\frac{r_{2}-r_{1}}{100})$$

### 3.1. Comparative Analysis of Cone Angle Calculation

**Figure 9:**
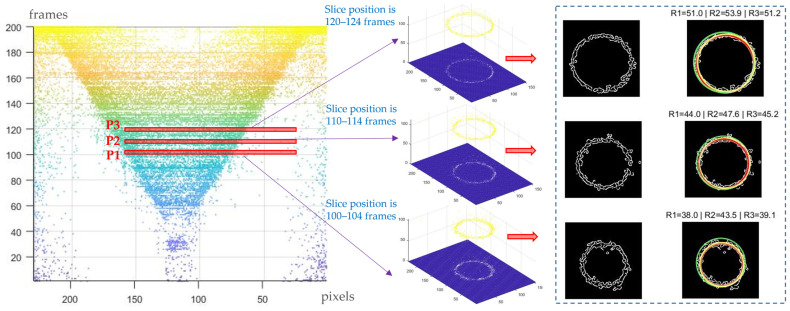
Slice circle fitting of fuel nozzle No. 1 (red line represents least squares method, green line represents RANSAC, and yellow line represents RC–RANSAC, the arrow indicates the process from the original slice to binary edge points and then to circle fitting).

When the magnification of the microscope was set to 3×, a series of images of the heat-treated fuel nozzle No. 1 inner hole were collected, as shown in Figure 9. Point cloud slices were selected at three positions, each with a thickness of four frames and a slice distance of 10 original frames. Circle fitting was performed using the least squares method, RANSAC, and RC-RANSAC. For point cloud slice P1, with positions ranging from 100 to 104 frames, the radii of the fitted circles were 38.0, 43.5 and 39.1 pixels. For point cloud slice P2, with positions ranging from 110 to 114 frames, the radii of the fitted circles were 44.0, 47.6 and 45.2 pixels. For point cloud slice P3, with positions ranging from 120 to 124 frames, the radii of the fitted circles were 51.0, 53.9 and 51.2 pixels.

In Figure 9, the green fitting line represents the RANSAC, the red fitting line corresponds to the least squares method, and the yellow fitting line denotes the RC-RANSAC method. Among the methods evaluated, RC-RANSAC produced the most accurate circle fitting. The runtime of the three algorithms are 2.5891 s, 2.6344 s, and 2.8074 s respectively. The computational efficiency of the three methods is not significantly different.

When using the least squares method for circle fitting at two slice positions, the radii of the fitted circles were 51 and 44 pixels, and the inclination angle was calculated as 52.8° according to Equation (12). Consequently, the inner hole conical angle β was determined as 105.6°. The circular fitting data for fuel injector No. 1’s workpiece is shown in Table 1.

Comparison of the differences between slices: The least squares method yielded radius differences of 6.0 and 7.0, the RANSAC resulted in 4.2 and 6.3, while the RC-RANSAC produced 6.1 and 6.0.

Comparison of cone angles: The least squares method resulted in conical angles of 96.9° and 105.6°. The RANSAC yielded 76.6° and 99.7°. The RC-RANSAC produced 97.9° and 96.9°. The radius differences of the least squares method are centered, with a conical angle difference of 8.7° between different slices. The RANSAC exhibits poor stability, with a conical angle difference of 23.1°. The improvement method proposed in this article yields the smallest deviation with a conical angle deviation of 1.0° between slices. So, the RC-RANSAC method led to the best circle fitting results.

**Figure 10:**
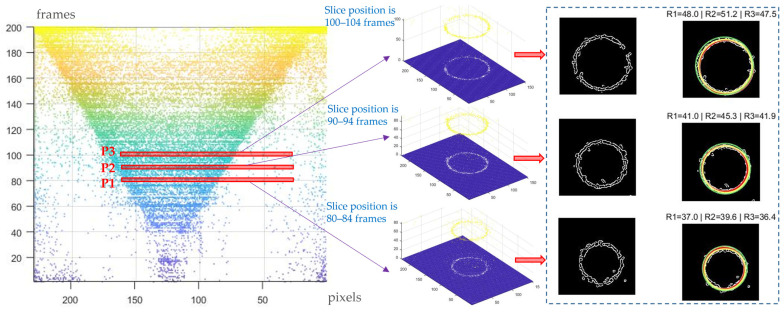
Slice circle fitting of fuel nozzle No. 2 (red line represents least squares method, green line represents RANSAC, and yellow line represents RC-RANSAC, the arrow indicates the process from the original slice to binary edge points and then to circle fitting).

When the magnification of the microscope lens is set to 3×, sequence images of the inlet inner hole of the fuel nozzle No. 2 after thermal treatment were collected, as shown in Figure 10. Sliced point clouds at three selected positions were taken, each with a thickness of 4. The circles were fitted using three circle fitting methods. When the P1 position of the point cloud slice is between 80 and 84 frames, the radius sizes of the circle fitting are 37.0, 39.6 and 36.4 pixels. The position of point cloud slice P2 slice is 90–94 frames, and the radius sizes of circle fitting are 41.0, 45.3 and 41.9 pixels. When the P3 position of the point cloud slice is between 100 and 104 frames, the radius sizes of the circle fitting are 48.0, 51.2 and 47.5 pixels. The circular fitting data for fuel injector No. 1’s workpiece is shown in Table 2.

Comparison of the differences between slices: The least squares method yielded differences of 4.0 and 7.0, while the RANSAC resulted in differences of 5.7 and 5.9. The RC-RANSAC method yielded differences of 5.5 and 5.6, respectively.

**Figure 11:**
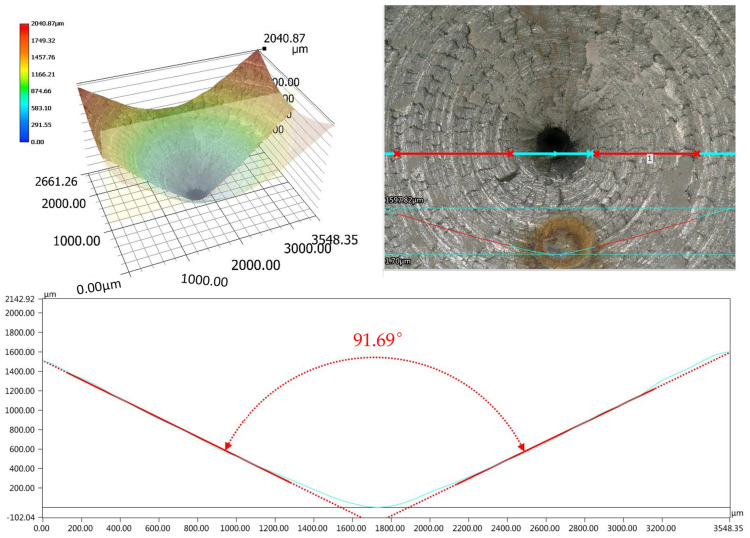
Measurement results of Keyence VHX−6000. (The number 1 represents detecting the taper of this section, the red lines indicated the detected area).

Comparison of cone angles: The least squares method produced cone angles of 73.9° and 97.3°. The RANSAC yielded cone angles of 94.0° and 96.0°. The RC-RANSAC resulted in cone angles of 92.0° and 93.0°. The radius difference deviation of the least squares method is relatively large, with a cone angle difference of 23.4° between different slices. The RANSAC exhibits better stability, with a smaller cone angle difference of 2°. The RC-RANSAC produces the smallest radius differences, with a cone angle deviation of 1° between slices. As shown in Figure 11, the cone angle of the inner hole measured by the Keyence ultra-depth platform VHX6000 (Keyence Corporation, Osaka, Japan) is 91.69°. The measurement error between the method described in this article and the Keyence ultra-depth detection is 0.8°. Therefore, among the three algorithms, the RC-RANSAC method yields the best circle fitting results.

### 3.2. Analysis of Circle Fitting Accuracy

In this study, the performance of different methods is evaluated using residual standard deviation (σ), root mean square error (RMSE), and inlier ratio (ε). The definitions of these metrics are as follows:

(13) $$\sigma =\sqrt{\frac{1}{N}\sum_{i=1}^{N}{\left(r_{i}-\bar{r}\right)}^{2}}$$

(14) $$RMSE=\sqrt{\frac{1}{N}\sum_{i=1}^{N}r_{i}^{2}}$$

(15) $$\varepsilon =N_{inlier}/N$$

**Figure 12:**
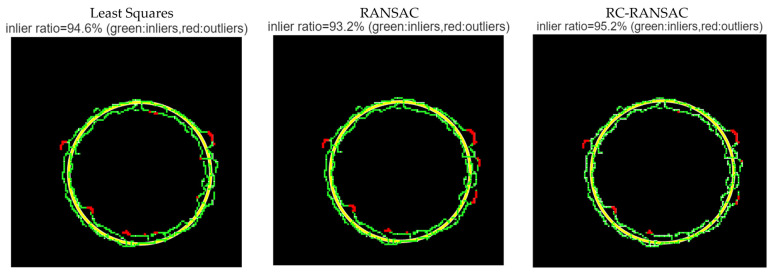
Comparison of interior point rates in circle fitting. (The yellow circle is the fitting circle).

Taking the inlet point cloud of the No. 1 fuel nozzle workpiece as an example, the point cloud with slice positions of 90–94 frames was binarized and subjected to circle fitting. The threshold for selecting the inner point ratio was 5 pixels, and the parameters were calculated as shown in Table 3. The inlier ratios of the three methods are shown in Figure 12.

To compare the performance of least squares (LS), RANSAC, and RC-RANSAC circle fitting algorithms, the residual standard deviation (σ), root mean square error (RMSE), and inlier ratio are adopted as evaluation metrics. Experimental results show that RC-RANSAC achieves the smallest σ (2.8260 px) and RMSE (2.8256 px), along with the highest inlier ratio (95.2%), demonstrating its superior robustness against outliers and minimal fitting deviation. Conventional RANSAC yields the largest σ (2.8849 px) and RMSE (2.8776 px) and the lowest inlier ratio (93.2%), indicating that the random sampling strategy fails to consistently converge to the global optimum. Although LS lacks an explicit outlier rejection mechanism, it outperforms conventional RANSAC in this dataset due to the relatively low outlier proportion, achieving an RMSE of 2.8554 px, yet is still inferior to RC-RANSAC. Considering an average fitted radius of approximately 41.6 px, the relative error is about 5.3%, reflecting limited edge localization precision of the original image. In summary, RC-RANSAC is the most favorable fitting strategy among the three; further improvement (e.g., RMSE < 1 px) would require subpixel edge extraction techniques.

## 4. Discussion

In this study, three circle fitting algorithms—least squares, conventional RANSAC, and the proposed RC-RANSAC—were applied to point cloud slice data of the inner holes of heat-treated fuel nozzles No. 1 and No. 2. Algorithm performance was systematically evaluated from four aspects: fitting radius consistency, inter-slice radius difference, taper angle stability, and comparison with Keyence ultra-depth measurements. To further quantify fitting quality, three metrics were introduced: residual standard deviation (σ), root mean square error (RMSE), and inlier ratio (ε).

Existing guided or constrained RANSAC methods (e.g., those based on geometric priors, local optimization, or sequential verification) typically improve sampling efficiency or model accuracy by introducing additional heuristics, often relying on user-defined parameters or strong prior assumptions about the target shape (such as curvature ranges or point-pair distances). In contrast, the proposed RC-RANSAC adopts a much lighter yet effective spatial constraint: the point cloud is divided into eight radial sectors according to the polar angle, and three points are forcibly sampled from three different sectors to compute circle parameters. This strategy inherently ensures spatial dispersion of the sampled points without requiring any prior geometric information (e.g., radius estimation or initial center guess), thereby significantly reducing the probability of collinear or overly concentrated triplets that lead to ill-conditioned models. Compared to existing approaches, the main distinctions of RC-RANSAC are:

(1) No shape priors are needed, making it highly adaptive.
(2) Computational overhead is minimal, involving only one angle calculation and sector assignment.
(3) Sampling interpretability—the sector division directly corresponds to the geometric orientation of the circle, facilitating engineering tuning. Experimental results confirm that RC-RANSAC outperforms both conventional RANSAC and least squares in fitting accuracy and robustness, with no significant increase in runtime, validating the effectiveness of this spatial constraint strategy.

Taking slice P2 (frames 110–114) of workpiece No. 1 as an example, RC-RANSAC achieved σ = 2.8260 px, RMSE = 2.8256 px, and ε = 95.2%, all superior to those of least squares (σ = 2.8492, RMSE = 2.8554, ε = 94.6%) and conventional RANSAC (σ = 2.8849, RMSE = 2.8776, ε = 93.2%). This demonstrates that RC-RANSAC has the strongest robustness against outliers, producing fitted circles that best match the true edge profile. The suboptimal performance of conventional RANSAC can be attributed to the inefficiency of its random sampling strategy, especially when the point cloud density is uneven, where samples are more likely to be drawn from locally dense regions, leading to model deviation.

For workpiece No. 1, although RC-RANSAC produced slightly larger inter-slice radius differences (6.1 and 6.0 pixels) compared to RANSAC (4.2 and 6.3 pixels), its taper angle deviation between slices was only 1.0°, far smaller than those of least squares (8.7°) and RANSAC (23.1°).

For workpiece No. 2, RC-RANSAC also achieved a taper angle deviation of 1.0°, outperforming least squares (23.4°) and RANSAC (2.0°).

These results indicate that RC-RANSAC provides the best cross-slice consistency in taper angle estimation. Even when local radius differences are not minimal, its robust center localization ensures stable taper estimates. In contrast, RANSAC exhibits large taper fluctuations (e.g., 23.1° for workpiece No. 1), reflecting poor consistency across slices—further evidence of the randomness inherent in conventional RANSAC due to the lack of spatial constraints.

For workpiece No. 2, the Keyence-measured taper angle was 91.69°. RC-RANSAC produced estimated taper angles of 92.0° (based on P2/P3 slices) and 93.0° (based on other slice combinations), yielding a measurement error of 0.8°. In contrast, the least squares method gave an error of 5.6°, and RANSAC gave an error of 2.3°. The close agreement between RC-RANSAC and Keyence validates the effectiveness of the proposed method for industrial inspection.

The runtimes of the three algorithms were 2.59 s, 2.63 s, and 2.81 s, respectively, showing no significant difference. This indicates that RC-RANSAC improves accuracy without introducing substantial computational overhead. The overhead of sector division and angle calculation is negligible, so the time complexity of RC-RANSAC remains comparable to that of conventional RANSAC.

The current RMSE of approximately 2.8 px corresponds to a taper error of about 0.3–0.5°, which may still affect precision assembly. One contributing factor, beyond raw image noise and edge detection errors, is that setting the point cloud slice thickness to four frames may generate excessive redundant edge points after binarization. These additional edges introduce local irregularities, thereby degrading circle fitting quality and increasing RMSE. To further reduce RMSE below 1 px, it is recommended to incorporate subpixel edge extraction techniques and optimize the slice thickness parameter (e.g., trying smaller or adaptive thickness). Furthermore, future research should expand the sample size and explore more advanced segmentation algorithms, such as deep learning, to handle complex scenarios. Although RC-RANSAC excels at dispersed sampling, when the point cloud becomes extremely sparse or some radial sectors are empty, a sector-merging strategy should be introduced—this is also a direction for further improvement.

## 5. Conclusions

This paper utilizes the principle of microscopic depth of field to obtain evenly spaced sequential images of the inlet hole of a fuel injector nozzle. A sharpness evaluation operator is then used to extract the initial point cloud data. A statistical outlier filtering method is applied to remove redundant data from the initial point cloud, and uniform voxel down sampling is used to simplify the point cloud data. The preprocessed point cloud data eliminates noise while retaining geometric features. The point cloud slicing method is employed to measure the conical angle of the fuel injector’s inner hole. Initially, point cloud slices with a thickness of 4 are selected at several evenly spaced positions, and the point cloud slices are converted from 3D points to 2D images. Simultaneously, the Canny edge detection method is used to extract the edge coordinates of the 2D images. Then the least squares, RANSAC, and RC-RANSAC methods are applied to fit circles to the sliced data. The experimental results show that the robust RANSAC method provides the best circle fitting results. This paper still has some limitations, and future work can increase the number of measurement samples and include a comparative analysis with deep learning algorithms, simultaneously applying laboratory research to online monitoring lines to verify the real-time detection efficiency of the method. Several limitations of this study should be acknowledged, including the fixed slice thickness (four frames) which may introduce redundant edges, and the relatively small sample size. Future work will increase the number of measurement samples, incorporate deep learning-based segmentation for complex scenarios, and deploy the method on online monitoring lines to verify real-time detection efficiency. In conclusion, RC-RANSAC offers a prior-free, efficient solution for automated taper measurement of fuel injector inner holes.

## Figures and Tables

**Table 1:** Measurement results of different algorithms for fuel nozzle No. 1.

Method	P1 Radius (Pixels)	P2 Radius (Pixels)	P3 Radius (Pixels)	Radius Difference (Pixels) (P1–P2)	Radius Difference (Pixels) (P2–P3)	Taper Angle (°)
Least Squares	38.0	44.0	51.0	6.0	7.0	$\beta_{12}$ = 96.9 $\beta_{23}$ = 105.6
RANSAC	43.5	47.6	53.9	4.1	6.3	$\beta_{12}$ = 76.6 $\beta_{23}$ = 99.7
RC-RANSAC	39.1	45.2	51.2	6.1	6.0	$\beta_{12}$ = 97.9 $\beta_{23}$ = 96.9

**Table 2:** Measurement results of different algorithms for fuel nozzle No. 2.

Method	P1 Radius (Pixels)	P2 Radius (Pixels)	P3 Radius (Pixels)	Radius Difference (Pixels) (P1–P2)	Radius Difference (Pixels) (P2–P3)	Taper Angle (°)
Least Squares	37.0	41.0	48.0	4.0	7.0	$\beta_{12}$ = 73.9 $\beta_{23}$ = 97.3
RANSAC	39.6	45.3	51.2	5.7	5.9	$\beta_{12}$ = 94.0 $\beta_{23}$ = 96.0
RC-RANSAC	36.4	41.9	47.5	5.5	5.6	$\beta_{12}$ = 92.0 $\beta_{23}$ = 93.0

**Table 3:** Calculation of circle fitting parameters.

Method	σ (Pixels)	RMSE (Pixels)	Inlier Ratio (%)	Fitting Radius (Pixels)
Least Squares	2.8492	2.8554	94.6	41.4449
RANSAC	2.8849	2.8776	93.2	41.5699
RC-RANSAC	2.8260	2.8256	95.2	41.7560

## Data Availability

The original contributions presented in this study are included in the article. Further inquiries can be directed to the corresponding author.
